# The relationship between local area labor market conditions and the use of Veterans Affairs health services

**DOI:** 10.1186/1472-6963-13-96

**Published:** 2013-03-13

**Authors:** Edwin S Wong, Chuan-Fen Liu

**Affiliations:** 1 Northwest Center for Outcomes Research in Older Adults, VA Puget Sound Health Care System, 1100 Olive Way, Suite 1400, Seattle, WA 98101, USA; 2 Department of Health Services, School of Public Health and Community Medicine, University of Washington, Box 357660, Seattle, WA 98195, USA

**Keywords:** Health services, Utilization, Veterans, Socioeconomic factors

## Abstract

**Background:**

In the U.S., economic conditions are intertwined with labor market decisions, access to health care, health care utilization and health outcomes. The Veterans Affairs (VA) health care system has served as a safety net provider by supplying free or reduced cost care to qualifying veterans. This study examines whether local area labor market conditions, measured using county-level unemployment rates, influence whether veterans obtain health care from the VA.

**Methods:**

We used survey data from the Behavioral Risk Factor Surveillance System in years 2000, 2003 and 2004 to construct a random sample of 73,964 respondents self-identified as veterans. VA health service utilization was defined as whether veterans received all, some or no care from the VA. Hierarchical ordered logistic regression was used to address unobserved state and county random effects while adjusting for individual characteristics. Local area labor market conditions were defined as the average 12-month unemployment rate in veterans’ county of residence.

**Results:**

The mean unemployment rate for veterans receiving all, some and no care was 5.56%, 5.37% and 5.24%, respectively. After covariate adjustment, a one percentage point increase in the unemployment rate in a veteran’s county of residence was associated with an increase in the probability of receiving all care (0.34%, p-value = 0.056) or some care (0.29%, p-value = 0.023) from the VA.

**Conclusions:**

Our findings suggest that the important role of the VA in providing health care services to veterans is magnified in locations with high unemployment.

## Background

In the United States (U.S.), economic conditions are intertwined with labor market decisions, access to health care, health care utilization and expenditures, and health outcomes because of employment-based health insurance. During the most recent national recession, the percentage of Americans with employer-based health insurance obtained directly or through a parent or spouse decreased from 59.3% (177.4 million) in 2007 to 55.8% (169.7 million) in 2009. During the same period, the number uninsured increased by 5 million and the number covered by Medicaid increased by 8.2 million [1]. When economic conditions are poor and unemployment is high, diminished access to care results from loss of health insurance, but may also arise from reductions in wealth resources and an individual’s ability to pay for health care. Therefore, safety net systems, such as state Medicaid programs, are important sources of health care services during difficult economic periods when labor market conditions are weak.

The Veterans Affairs (VA) health care system has served as a health care safety net [2-4] for veterans by providing free or reduced cost care based on the level of military service-related disability, or by satisfying a financial means test. VA is the largest integrated health care system in the U.S. with a health care budget of $52 billion in 2011. The VA system provided health care to about 6 million patients at 152 medical centers and more than 800 community-based outpatient clinics in 2011 [5]. Between 1999 and 2010, VA enrollment increased from 4.2 million to 8.3 million [6]. VA is widely acknowledged as providing quality of care equal to or better than care provided through Medicare or private insurance [7,8]. VA serves a unique population with patients who are sicker, have low incomes and are more socially disadvantaged compared to general populations. VA also provides services to meet veterans’ special health care needs, such as spinal cord injuries, amputations and serious mental illness.

The minimum requirement for receiving health benefits from VA is veteran classification, defined as previous service in the active military, naval or air service. Veteran status also requires discharge from service for any reason other than dishonorable. After January 17, 2003, VA restricted new enrollment to veterans with service connected disabilities or conditions, who satisfy a financial means test or meet special criteria (i.e. prisoner of war) [9]. All other veterans applying for VA care are placed on a waitlist. Once enrolled, veterans have access to all VA health services. Veterans eligible for VA health benefits are also free to enroll in other health plans and obtain care from non-VA sources.

Veterans receive care directly related to all service connected disabilities free of charge. For all other non-service connected care, veterans’ level of cost sharing is determined by priority group classification, ranging from 1 (highest priority) through 8 (lowest priority). Priority group classification is based on service connected disability and financial means, but may also be determined by special circumstances such as exposure to Agent Orange while serving in Vietnam between 1962 and 1965. Among those exempt from all copayments are veterans with service-connected disabilities rated at least 50% disabling (priority 1). In addition, priority 5 veterans, classified as those living below the national means test threshold, are exempt from all copayments for medical treatment. In fiscal year (FY) 2011, veterans in priority groups 1 and 5 constituted 14.9% and 26.7% of all VA enrollees [10]. Veterans in other priority groups may qualify for free care based on other factors such as catastrophic disability. All other veterans are subject to copayments for some or all of their VA care. VA also has programs that provide health care when individuals face financial hardship including periods after the loss of a job or a significant decrease in household income.

The objective of this paper is to examine the relationship between local area labor market conditions and patterns of utilization of VA health care services among veterans using a repeated cross-sectional sample from the Behavioral Risk Factor Surveillance System (BRFSS). As in previous studies [11,12], local labor market conditions were measured using county-level unemployment rates over a retrospective twelve-month period. This study used data from a national sample of adults to present an overall picture of health service use from a full sample of veterans, including those not enrolled in the VA and those with no VA health service use to provide important policy information on potential impacts of unemployment rates on the use of VA services among all veterans. To our knowledge, this is the first study to measure the impact of labor market conditions on VA specific health service use. As the largest integrated health care system in the U.S. charged with serving a continually growing veteran population, understanding factors affecting veterans' reliance on VA care is important for determining efficient budget levels, while maintaining high quality of care.

## Methods

### Conceptual model

We analyzed the use of VA care within the context of the Behavioral Model of Health Services Use [13-15]. The Behavioral Model posits that three sets of population characteristics influence health service use. Predisposing characteristics such as age, gender and marital status affect factors that enable or impede the use of health services. Correspondingly, enabling factors such as income, insurance and social support influence need for care. In conjunction with predisposing, enabling and health need factors, the Behavioral Model also recognizes personal health practices as a determinant of formal health service use [14,16]. Prior studies have found that veterans with greater reliance on VA care were between 50–64 years old, black, single, uninsured, classified in high VA priority groups and had lower income [2,10,17].

We hypothesized that local area labor market conditions affect individuals’ use of VA health services through enabling factors. Specifically, individuals residing in geographical areas of high unemployment were less likely to be employed and have employer-sponsored health insurance, earn lower income and subsequently more likely to enroll in the VA and use VA health benefits. Local area labor market conditions, as a measure of enabling resources, are important to VA policymakers because they are more readily available relative to other individual level measures such as income or health insurance status.

### Data

The sample of veterans for this study was drawn from BRFSS, which is a state-based health survey with the purpose of collecting information on health risk behaviors, practices and health care access [18]. BRFSS data is publically available and collected annually through phone interviews. There is a core survey asked to respondents in all 50 states. Additionally, each state may ask supplemental questions. The content of surveys is jointly determined by the Centers for Disease Control and state health departments, varying by year and is dependent on the data needs of state health departments. Repeated cross-sectional data used in this study were from the calendar years 2000, 2003 and 2004. Questions regarding VA health service use were discontinued after 2004. BRFSS data has been previously used to examine issues related to VA use among veterans [19-21].

Veterans identified in BRFSS data were linked with county-level unemployment data from the Area Resource File (ARF) [22]. Geographical VA facility data were obtained from the Veterans Administration Site Tracking System (VAST) [23].

### Study sample

We derived a sample of respondents identified as veterans in the calendar years 2000, 2003 and 2004. Veterans were identified in BRFSS as those who ever served in active duty in the U.S. armed forces. There were 105,630 respondents that indicated veteran status. We excluded respondents who were missing data for county of residence (19,266 subjects) and other covariate data (12,401 subjects).

### VA health service utilization

The primary outcome variable was self-reported use of VA health care services. All respondents identified as veterans were asked whether they received health care at VA facilities during the previous 12 months. Those receiving care were then asked whether VA health care constituted all or some of their total health care utilization. We categorized the outcome variable into three groups: no VA use, some VA care, and all VA care.

### Independent variables

The primary explanatory variable of interest was local area labor market conditions, which we specifically measured using county-level unemployment rates from ARF data. County unemployment rates were calculated monthly and were defined as the number of unemployed persons divided by the civilian labor force times 100. A person was defined as unemployed in a given month if he or she was not employed in the week that included the 12^th^ day of the month, was available for work and made specific efforts to find work during the prior four weeks. The unemployment rate corresponding to a veteran was calculated as the average unemployment rate in the county of residence over the 12 months prior to survey completion. The unemployment rate variable was constructed in this manner in order to coincide with the 12 month retrospective measurement of VA health service use. Because the administration of BRFSS is staggered throughout the calendar year, individuals from the same survey year and living in the same county may have a different average unemployment rate. This individual variation in the unemployment rate within counties provides the ability to disentangle the effect of local area labor market conditions from other unobserved time invariant county factors, which is an approach used previously in other studies [11,12].

To estimate the impact of county-level unemployment as a measure of enabling resources, we adjusted for a number of other characteristics including individual demographics (age, gender, race, marital status, education, number of children under 18), behavioral factors (exercise behavior and smoking status) and health status (body mass index (BMI), self reported health status, numbers of poor physical and mental health days in the last year) from BFRSS data. We included BMI because recent studies have identified associations between obesity and health service use [24,25]. Because eligibility for Medicare may result in differences in the effect of the unemployment rate across age, we included a variable indicating if a veteran was age 65 or over and the interaction with the unemployment rate in the set of individual characteristics. To control for changes in VA enrollment policy in 2003, we included indicator variables for calendar year. Finally, because prior research suggests access to VA care is an important determinant of VA health service use [26], we also included the number of VA health care facilities in the county of residence during the previous year.

### Statistical analysis

We used hierarchical ordered logistic regression to estimate the relationship between local area labor market conditions and the use of VA health care services among veterans. The dependent variable measures the use of VA health care services and was an ordered categorical response (all, some or no health care from the VA). The underlying latent variable was the percentage of health care that is obtained from the VA. The specific model (model 1) we estimated was:

(1) $$\text{UTI}L^{*}{}_{\mathrm{ij}}=\alpha E_{\mathrm{ij}}+X_{\mathrm{ij}}\beta +Z_{\mathrm{ij}}\gamma +\varepsilon_{\mathrm{ij}}$$

where UTIL*_ij_ was the latent variable for VA health care utilization for individual *i* in county *j*, E_*ij*_ was the unemployment rate, X_*ij*_ was a vector of individual characteristics, Z_*ij*_ was a vector of random-effects parameters and ε_*ij*_ was the idiosyncratic error term. We included random intercept terms for state and county (nested within state) to account for the clustering of veterans and to account for other time invariant characteristics that potentially affect utilization, such as non-VA local area healthcare resources.

Marginal effects obtained from the base model (equation 1) were used to assess the relationship between VA health care service use and the unemployment rate. Marginal effects relate the change in the probability of a particular outcome (receiving all, some, or no care at VA facilities) resulting from a one percentage point increase in the unemployment rate. The marginal effect for all VA care was computed as

(2) $$\begin{array}{l} \frac{\partial \mathrm{Pr}\left(\text{All}\;\mathrm{VA}\;\text{Care}\right)}{\partial E_{\mathrm{ij}}}=\hat{\alpha }*\hat{\text{P}}\text{r}\left(\text{All}\;\mathrm{VA}\;\text{Care}\right)\\ \;*\left(1-\hat{\text{P}}\text{r}\left(\text{All}\;\mathrm{VA}\;\text{Care}\right)\right) \end{array}$$

where *â* was the estimated ordered logistic coefficient for unemployment in equation 1 and $\hat{\text{P}}\text{r}\left(\text{All}\;\mathrm{VA}\;\text{Care}\right)$ was the adjusted probability of receiving all care from the VA.

Marginal effects were computed for all respondents, and averaged across the respective sample of respondents below age 65 and age 65 and above. For respondents age 65 and above, *â* in equation 2 was replaced by the sum of *â* and the estimated ordered logistic regression coefficient for the unemployment-age-over-65 interaction. Analogous marginal effects were computed for the probability of veterans receiving some or no care at the VA, respectively. We also examined the interaction between age group and the county-level unemployment rate, as outlined in a prior study [27]. Specifically, we compared unemployment rate marginal effects across age groups, and found the differences were statistically significant.

Standard errors for parameter estimates in equation (1) were estimated using the Huber-White sandwich estimator. The delta method was then used to compute standard error estimates for marginal effects. The county-level intraclass correlation coefficient (ICC) was computed by dividing the county-level variance estimate by the sum of the variance estimates from all levels (respondent, county and state) [28]. The state level ICC was calculated in an analogous manner. All statistical analyses were completed using the STATA statistical software (Version 11; College Station, TX). The GLLAMM procedure was used to perform hierarchical ordered logistic regression. A nominal p-value of 0.05 was used to assess statistical significance.

To further examine whether local area labor market conditions affect VA health service use through enabling resources, we performed two additional analyses. First, to examine the extent to which veterans may increase VA health service use because of loss of health insurance, we stratified the analysis by employment status at the individual level, assuming those who were employed were also more likely to be covered by health insurance (model 2). We did not use data regarding health care coverage in BRFSS because these survey questions did not distinguish between private insurance and other government plans such as Medicare or VA. Second, we examined whether the effect of local area labor market conditions was mediated by income in the subsample of employed veterans (model 3). In this model, we included income as an adjustment variable. Missing data for income (7,499 observations) and employment status at the individual level (92 observations) were imputed using multiple imputation [29]. Survey weighting was not applied in our analysis because weights are required for each level of the hierarchical model and were not available. The BRFSS sampling stratum (area code/prefix) did not correspond to the county and level units in our hierarchical analysis.

## Results

### Descriptive statistics

This study included 73,964 veterans in the U.S. during calendar years 2000, 2003 or 2004 (Table 1). The characteristics of our study sample corresponded to demographics of the U.S. veteran population [30]. Specifically, the majority of respondents were male and married, and the mean age was approximately 60 years of age. The proportion of veterans who were white was larger compared to the U.S. general population. The proportion of employed veterans was 40.3% overall and was highest in counties with the lowest unemployment rates. Income levels were skewed toward the lower income categories. Over 81% of veterans reported health status as being good or better. On average, veterans reported 4.15 physical poor health days and 2.56 mental poor health days per year. The proportion of veterans receiving all care from the VA was higher in counties where the unemployment was higher (chi-square trend = 28.989, p-value < 0.001). Veterans age 65 and above were more likely to obtain at least some their care from the VA than veterans below age 65 (Table 2). The difference was largely due veterans over 65 being much more likely to obtain some care from the VA (13.69% vs. 6.81%).

**Table 1:** Demographic characteristics of BRFSS veterans sample

		**Unemployment quartile**			
	**Total**	**1**^**st **^**(0.70–3.85)**	**2**^**nd **^**(3.86–4.89)**	**3**^**rd **^**(4.90–6.33)**	**4**^**th **^**(6.33–29.88)**
**Observations**	73,964	18,596	18,169	18,662	18,537
**Demographics (% or mean)**					
Male (%)	92.5	92.3	92.3	93.0	92.3
White (%)	88.5	92.3	88.8	87.7	85.2
Black (%)	6.7	4.4	5.8	8.0	8.4
Age in years (SD)	59.27 (14.76)	58.33 (14.92)	59.41 (14.69)	59.86 (14.66)	59.48 (14.71)
Over 65 (%)	39.7	37.4	39.8	41.2	40.4
Married (%)	63.7	65.3	64.6	63.1	61.8
# Children under 18 (SD)	0.37 (0.88)	0.38 (0.90)	0.37 (0.86)	0.36 (0.87)	0.37 (0.89)
*Education*					
< High school (%)	6.6	6.0	6.2	6.4	7.6
High school (%)	29.2	28.1	28.9	29.4	30.5
Attended college (%)	29.7	29.0	29.3	29.6	30.8
Completed college (%)	34.6	37.0	35.5	34.7	31.2
Employed (%)	40.3	44.5	41.2	38.8	36.8
*Income*					
< $25,000 (%)	24.5	22.2	22.5	24.7	28.8
> = $25,000–$50,000 (%)	36.0	35.6	35.6	35.9	36.8
> = $50,000–$75,000 (%)	18.7	19.5	19.2	18.9	17.1
> = $75,000 (%)	20.8	22.7	22.7	20.6	17.3
**Behavioral characteristics**					
Vigorous exercise (%)	76.7	77.0	77.1	76.9	75.9
*Smoking status*					
Current smoker (%)	21.2	20.7	20.6	21.3	22.4
Former smoker (%)	44.8	44.3	45.3	45.5	44.1
Never smoked (%)	33.9	35.0	34.0	33.2	33.5
*Self reported health*					
Excellent (%)	18.7	20.3	19.1	18.3	17.2
Very Good (%)	31.2	33.1	32.0	30.3	29.5
Good (%)	31.2	30.2	30.9	32.1	31.7
Fair (%)	12.9	11.2	12.6	13.1	14.6
Poor (%)	5.9	5.1	5.5	6.1	7.0
# Physical poor health days (SD)	4.15 (8.96)	3.73 (8.51)	4.00 (8.82)	4.26 (9.07)	4.62 (9.41)
# Mental poor health days (SD)	2.56 (7.05)	2.29 (6.61)	2.42 (6.89)	2.67 (7.21)	2.88 (7.44)
*Body mass index* (*Category*)					
Underweight (%)	0.7	0.7	0.7	0.7	0.7
Normal (%)	28.7	30.0	28.8	28.5	27.5
Overweight (%)	47.0	47.1	47.1	46.9	47.0
Obese (30 < = BMI < 40) (%)	21.9	20.7	21.9	22.2	22.9
Severely obese					
(BMI > = 40) (%)	1.6	1.5	1.5	1.6	1.8
**County variables**					
# VA facilities (SD)	0.89 (1.12)	0.69 (0.85)	0.86 (0.88)	1.08 (1.18)	0.94 (1.43)
Unemployment rate (SD)	5.26 (2.20)	3.02 (0.59)	4.36 (0.29)	5.55 (0.41)	8.08 (2.20)
**Time effects**					
Year =2000 (%)	22.7	51.4	20.9	9.5	8.9
Year =2003 (%)	34.6	20.2	35.4	39.9	42.9
Year = 2004 (%)	42.7	28.5	43.6	50.5	48.2
**Receiving VA care**					
All health care from VA (%)	7.9	7.2	7.8	7.8	8.8
Some health care from VA (%)	9.5	8.8	9.5	10.3	9.5
No health care from VA (%)	82.6	83.9	82.7	81.9	81.8

BMI = Body Mass Index; SD = Standard Deviation.

**Table 2:** Use of VA health services by age group (under 65 versus over 65)

	**Amount of health care obtained from VA**			
	**All**	**Some**	**None**	**Total**
	**(n = 5,835)**	**(n = 7,055)**	**(n = 61,074)**	**(n = 73,964)**
Age < 65	8.00%	6.81%	85.19%	100%
(n = 44,599)				
Age > =65	7.72%	13.69%	78.59%	100%
(n = 29,365)				
Total	7.89%	9.54%	82.57%	100%
(n = 73,964)				
Chi-Square^1^	973.58			
P-Value	< 0.001			

^1^Test of the null hypothesis of no association between age group and the amount of VA care.

### Adjusted results

Table 3 presents average local area unemployment marginal effects, after adjusting for covariates, by age above or below 65 years old for all three model specifications. For the full sample from the base model (model 1), a one percentage point increase in the county-level unemployment rate was associated with a 0.63% decrease in the probability of receiving no care (p-value = 0.038, 95% Confidence Interval (CI), -1.22% to -0.03%), and a 0.29% increase in the probability of receiving some care from the VA (p-value = 0.023, 95% CI: 0.04% to 0.54%). The marginal effect for the probability of receiving all care from the VA was positive and approaching statistical significance (Marginal Effect (ME) = 0.34%, p-value = 0.056, 95% CI: -0.01% to 0.68%).

**Table 3:** County-level unemployment rate marginal effects

**Use of VA**	**Model 1**			**Model 2**				**Model 3**	
**Healthcare**				**Unemployed**		**Employed**			
	**Full sample**	**Age**	**Age**	**Age**	**Age**	**Age**	**Age**	**Age**	**Age**
		**> = 65**	**< 65**	**> = 65**	**< 65**	**> = 65**	**< 65**	**> = 65**	**< 65**
No VA Care (%)	-0.63*	-0.80*	-0.39*	-0.71*	-0.45**	-0.30	-0.10	-0.03	-0.01
(SE)	(0.30)	(0.25)	(0.18)	(0.30)	(0.16)	(0.40)	(0.13)	(0.10)	(0.06)
Some VA Care (%)	0.29*	0.11	0.21*	0.27***	0.24**	0.04	0.05	0.03	0.02
(SE)	(0.13)	(0.11)	(0.10)	(0.08)	(0.08)	(0.10)	(0.07)	(0.11)	(0.07)
All VA Care (%)	0.34	0.70	0.17*	0.44	0.21**	0.26	0.04	0.05	0.03
(SE)	(0.18)	(0.36)	(0.08)	(0.24)	(0.07)	(0.36)	(0.06)	(0.21)	(0.13)

Marginal effect estimates reflect percentage changes in the probability of receiving all, some or no healthcare from VA resulting from unit increase in the county unemployment rate.

Model 1: The baseline model including variables for county unemployment rate, age over 65 and the interaction between unemployment rate and age over 65.

Model 2: The baseline model stratified by individual employment status.

Model 3: The baseline mode also including individual income as an adjustment variable.

Statistical significance at the 5%(*), 1%(**) and 0.1%(***) level.

For model 1, among veterans who were under 65 years old, higher county-level unemployment was associated with an increase in the probability of receiving all (ME = 0.17%, p-value = 0.034, 95% CI: 0.01% to 0.33%) or some (ME = 0.21%, p-value = 0.028, 95% CI: 0.02% to 0.40%) care and a decrease in the probability of receiving no care (ME = -0.39%, p-value = 0.030, 95% CI: -0.73% to -0.04%) from the VA. For veterans age 65 and above, higher unemployment was also associated with a decrease in the probability of receiving no care (ME = -0.80%, p-value = 0.012, 95% CI: -1.43% to -0.18%) from the VA. We did not find a statistically significant association for the probability of receiving some care from the VA (ME = 0.11%, p = 0.34, 95% CI: -0.11% to 0.33%), however, the marginal effect for the probability of receiving all care was approaching statistical significance (ME = 0.70%, p-value = 0.054, 95% CI: -0.01% to 1.40%).

Estimates of the variance components for state (0.066, p-value < 0.001, 95% CI: 0.031 to 0.101) and county (0.102, p-value < 0.001, 95% CI: 0.080 to 0.123) were both statistically significant for model 1. Based on these covariance parameter estimates, the percent of variation in the likelihood of receiving all care from the VA explained by unobserved state and county-level effects was 2.94% and 1.91%, respectively.

After stratifying veterans by individual employment status (model 2), increases in the county-level unemployment rate were associated with increases in the probability of receiving all (ME = 0.21%, p-value = 0.003, 95% CI: 0.07% to 0.36% under age 65; ME = 0.44%, p-value = 0.065, 95% CI: -0.03% to 0.91% over age 65) or some (ME = 0.24%, p-value = 0.005, 95% CI: 0.07% to 0.41% under age 65; ME = 0.27%, p-value = 0.001, 95% CI: 0.11% to 0.43% over age 65) care from the VA, but were associated with a decreased probability of receiving no care from the VA (ME = -0.45%, p-value = 0.004, 95% CI: -0.76% to -0.15% under age 65; ME = -0.71%, p-value = 0.018, 95% CI: -1.30% to -0.12% over age 65) among unemployed veterans. However, among employed veterans, there were no significant associations between the county-level unemployment rate and use of VA care.

After adjusting for respondent income in the sample of employed veterans (model 3), we did not find a statistically significant association between the county-level unemployment rate and the probability of receiving no care (ME = -0.01%, p-value = 0.80, 95% CI: -0.13% to 0.10% under age 65; ME = -0.03%, p-value = 0.80, 95% CI: -0.20% to 0.17% age 65 and over) some care (ME = 0.02%, p-value = 0.80, 95% CI: -0.13% to 0.16% under age 65; ME = 0.03%, p-value = 0.80, 95% CI: -0.19% to 0.24% age 65 and over) and all care (ME = 0.03%, p-value = 0.80, 95% CI: -0.22% to 0.29% under age 65; ME = 0.05%, p-value = 0.80, 95% CI: -0.35% to 0.46% age 65 and over) from the VA. A sensitivity analysis estimating models 2 and 3 without missing data imputation produced similar results.

We further validated the impact of local area unemployment as a measure of enabling resources by assessing the relationship between VA health service use and veterans experiencing cost barriers to receiving care, defined as unable to see a doctor because of costs in the past 12 months. A higher percentage of veterans experiencing cost barriers received all or some of their care from the VA compared to those not reporting cost barriers (Table 4).

**Table 4:** VA health service use by cost barrier category

	**Amount of health care obtained from VA**			
**Cost barriers**	**All**	**Some**	**None**	**Total**
	**(n = 5,819)**	**(n = 7,045)**	**(n = 61,006)**	**(n = 73,870)**
No cost barriers	7.49%	9.42%	83.10%	100%
(n = 68,428)				
With cost barrier	12.81%	11.04%	76.15%	100%
(n = 5,442)				
Total	7.88%	9.54%	82.59%	100%
(n = 73,870)				
Chi-Square^1^	224.75			
P-Value	< 0.001			

^1^Test of the null hypothesis of no association between cost barriers and the amount of VA care.

## Discussion

This study examined the relationship between the use of VA health care services by veterans and labor market conditions in their county of residence, measured using the county-level unemployment rate. After controlling for individual covariates and random county and state effects, we found that poorer local area labor market conditions were associated with significant increases in the likelihood of VA health service use and significant decreases in the likelihood of receiving no VA care at all.

Our results are consistent with other prior studies finding an increased burden on public payers during periods of economic downturn [31-33]. During the most recent recession in 2009, Martin and colleagues found a decrease in private health insurance enrollment, growth in out-of-pocket spending and an increase in per enrollee Medicare spending growth [33]. In the general population, use of medical services (as measured by hospitalization and doctor visits) were also found to increase when the economy weakens [12]. These results were attributed to deteriorating health during times of low unemployment. Other studies have found increased mental health utilization including psychiatric emergency services [34] and admissions to mental health facilities for alcohol-related disorders [35].

We conducted several sensitivity analyses to provide evidence that poorer labor market conditions increase VA health service use through reductions in veterans’ enabling resources. Based on the stratification analysis by individual employment status, the results show similar significant marginal effects among veterans who were unemployed, which suggest that the increased use of VA health care was in part due to loss of employer sponsored health insurance. Furthermore, we adjusted for individual income in the sample of employed veterans and the results show income mediated the impact of the county-level unemployment rate on use of VA care. Finally, we found Veterans reporting cost barriers to receiving care were more likely to obtain at least some of their care from VA. Overall, our results suggest that the county-level unemployment rate is an important metric of veterans’ enabling resources and has impacts relevant to VA policymakers with regard to demand projections.

Intraclass correlation estimates for state and county random effects suggest that up to 5% of the variation in VA health service use is affected by local area resources. These estimates are at the upper range of values found in previous studies examining the correlation in utilization measures within geographical units [36-38]. State random effect estimates also suggest that variation in health policies across states, including Medicaid, is an important determinant of whether veterans use VA care.

Use of VA health services does not preclude veterans from enrolling in other health plans and obtaining care from other sources. In particular, nearly all Americans are eligible for health benefits from Medicare starting at age 65. Our study showing the association between local area unemployment rates and use of VA health services was stronger among veterans above age 65 compared to those under 65, reflects the importance of the VA even among Medicare eligible veterans. This result is consistent with prior findings showing a substantial number of veterans are dual users of VA and non-VA health services [39]. There are several possible reasons for this result. First, as the overall demand for health services increases with age, veterans may selectively choose to obtain some of their care from the VA. For example, veterans selectively seek mental health care in VA as such services with limited coverage by Medicare and other payers [17,40]. VA also provides services to meet veterans’ special health care needs, such as spinal cord injuries, amputations and post-traumatic stress disorder. Second, this finding may stem from the fact that many counties with high unemployment have persistent poverty [41]. Veterans in these counties would have lower accumulated wealth resources and retirement income making them more likely to qualify for VA care. Finally, some veterans age 65 and above may not receive Medicare benefits or are unable to pay expenses not covered by Medicare. A recent study found that over 80% of VA enrollees older than 65 years of age were covered by Medicare in 2011 [42]. Also, in our sample, 3.56% of Medicare eligible veterans in our sample reported cost barriers to obtaining care.

Collectively, our results suggest that veterans were more likely to shift care to the VA, potentially as a health care source of last resort in areas where labor market conditions are poor. However, a substantial number of veterans have access to other sources of health care, suggesting that VA use is a choice for many [17,40,43-45]. Our results showing that over 76% of veterans reporting cost barriers also do not receive any care from the VA suggest that these disadvantaged veterans may be receiving care from other safety net providers, such as Medicaid.

The VA healthcare system has traditionally served as a safety net provider [2-4]. Our findings provide evidence that the role of the VA is magnified in locations and during periods where unemployment is high. In times when the economy weakens, safety net facilities such as those in the VA are susceptible to budget cuts, which may undermine the ability to provide quality health services to eligible veterans who require care. Our results further indicate that the determination of the VA health care budget should weigh the impact of macroeconomic conditions in order to provide the highest quality of care while minimizing costs. Areas hardest hit by the business cycle may require the most funding to ensure quality. The estimated marginal effects, while small, translate into substantial costs given an increasingly large veteran population and the VA mandate of serving veterans for life. For example, in 2010, the veteran population in the United States was 22,568,578 and the average annual VA medical care expenditure per patient was $7,970 [46]. We estimated a 1% increase in the unemployment rate was associated with a 0.63% increase in the likelihood that a veteran would receive some or all care from the VA. Based on the 2010 veteran population, our estimated effect size translates into 142,182 additional patients at an additional cost of $1.1 billion.

This study has several limitations. First, unemployment was measured at the county level, which is the smallest geographical unit publicly available in BRFSS. It is possible that unemployment is not homogenous within a county. Also, respondents may be employed in a county adjacent to their county of residence. Therefore, the county-level unemployment rate may not reflect the true labor market conditions a respondent is subject to. Second, the available BRFSS data lack detailed VA specific characteristics, such enrollment priority groups and military service related disability previously shown to impact VA utilization [2]. Third, because of limitations in data, we were unable to use sampling weights to adjust for the BRFSS sampling design. As a result, if the sample of veterans captured in data is not a random sample of veterans in each county then our standard error estimates for unemployment may be underestimated. Finally, all data in BRFSS is self-reported and subject to recall bias.

## Conclusions

In conclusion, we found that veterans in the U.S. were more likely to use the VA health care system in locations where local unemployment rates are high. This increased use of VA care was due, at least in part due to reductions in the enabling resources of veterans. The impact of county-level labor market conditions on VA health service use was present among all veterans, including those eligible for Medicare. Overall, the results in this study point to the continued importance of VA as a health provider for veterans, particularly when the state of the economy is weak.

VA currently uses the Enrollee Health Care Projection model to produce annual budget estimates, which accounts for veterans’ age, gender, geographic distribution and reliance on VA care [6,47]. Our results suggest the inclusion of variables measuring economic conditions, such as unemployment rates, could improve the accuracy of budget projections used to inform VA policy. Accurate projections are important to ensure VA receives funding levels that balance veterans’ health needs and fiscally responsibility.

## Abbreviations

US: United States; VA: Department of Veterans Affairs; FY: Fiscal year; BRFSS: Behavioral Risk Factor Surveillance System; ARF: Area Resource File; VAST: VA Administration Site Tracking; BMI: Body mass index; ICC: Intraclass correlation; CI: Confidence interval; ME: Marginal effect.

## Competing interests

All authors do not report any conflicts of interest.

## Authors’ contributions

ESW conceived of the study, prepared the data for analysis, performed the statistical analyses and drafted the manuscript. Both authors participated in the design of the study, the analysis and interpretation of data and the critical revision of the manuscript for intellectual content. Both authors have read and approve the final manuscript.

## Authors’ information

ESW and CFL: Northwest Center for Outcomes Research in Older Adults, VA Puget Sound Health Care System, 1100 Olive Way, Suite 1400, Seattle, WA 98101 USA.

## Pre-publication history

The pre-publication history for this paper can be accessed here:

http://www.biomedcentral.com/1472-6963/13/96/prepub
